# Home noninvasive ventilation use in patients hospitalized with COPD

**DOI:** 10.1111/crj.13678

**Published:** 2023-07-31

**Authors:** Spyridon Fortis, Yubo Gao, Kelby Rewerts, Mary Vaughan Sarrazin, Peter J. Kaboli

**Affiliations:** ^1^ Veterans Rural Health Resource Center‐Iowa City, VA Office of Rural Health, and Center for Access and Delivery Research and Evaluation (CADRE) at the Iowa City VA Healthcare System Iowa City Iowa USA; ^2^ Department of Internal Medicine, Division of Pulmonary, Critical Care, and Occupational Medicine University of Iowa Roy J. and Lucille A. Carver College of Medicine Iowa City Iowa USA; ^3^ Department of Internal Medicine, Division of General Internal Medicine University of Iowa Roy J. and Lucille A. Carver College of Medicine Iowa City Iowa USA

**Keywords:** COPD, hypercapnia, noninvasive ventilation

## Abstract

**Introduction:**

The study objective was to estimate the prevalence of chronic hypercapnic respiratory failure (CHRF) and home noninvasive ventilation (NIV) use in a high‐risk population, individuals with a history of at least one COPD‐related hospitalizations.

**Methods:**

We retrospectively analyzed electronic medical record data of patients with at least one COPD‐related hospitalization between October 1, 2011, and September 30, 2017, to the Iowa City VA Medical Center. We excluded individuals with no obstructive ventilatory defect.

**Results:**

Of 186 patients, the overall prevalence of compensated hypercapnic respiratory failure (CompHRF), defined as PaCO_2_ > 45 mmHg with a pH = 7.35–7.45, was 52.7%, while the overall prevalence of home NIV was 4.3%. The prevalence of CompHRF was 43.6% and home NIV was 1.8% in those with one COPD‐related hospitalization. Among those with ≥4 COPD‐related hospitalizations, the prevalence of CompHRF was 77.8% (14 of 18), and home NIV was 11.1% (2 of 18).

**Conclusion:**

Approximately half of individuals with at least one COPD‐related hospitalization have CompHRF, but only 8.2% of those use home NIV. Future studies should estimate CHRF rates and the degree of underutilization of home NIV in larger multicenter samples.

## Graphical abstract

Approximately half of individuals with at least one COPD‐related hospitalization have hypercapnia, but only 8.2% of those use home noninvasive ventilation. Future studies should estimate chronic hypercapnic respiratory failure rates and the degree of underutilization of home noninvasive ventilation in larger multicenter samples and identify barriers and facilitators for home noninvasive ventilation use in patients with COPD‐related hospitalizations.

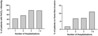

## 1 INTRODUCTION

Home noninvasive ventilation (NIV) in patients with chronic hypercapnic respiratory failure (CHRF) due to chronic obstructive pulmonary disease (COPD) reduces re‐admissions and mortality.^1^, ^2^, ^3^ Administrative data of patients hospitalized with COPD exacerbation showed that only 2.5% of them have received home bilevel positive airway pressure device or NIV devices.^4^ The CHRF and home NIV rates in COPD are understudied. This study objective was to estimate the prevalence of CHRF and home NIV use in a high‐risk population, individuals with a history of COPD‐related hospitalizations.

## 2 METHODS

We retrospectively analyzed electronic medical record data of individuals with at least one COPD‐related hospitalization between October 1, 2011, and September 30, 2017, to the Iowa City VA Medical Center. The Institutional Review Board and Research and Development Committee at the Iowa City VA [IRB 201712713] approved this study. COPD‐related hospitalizations were defined as hospitalizations using International Classification of Diseases codes (ICD‐CM)_._ ^5^

We excluded individuals with no available spirometry data or obstructive ventilatory defect. We defined obstructive ventilatory defect as a pre‐bronchodilator FEV_1_/FVC < 0.7 or FEV_1_% predicted<80% combined with a residual volume %predicted>120% or residual volume/total lung capacity>120%predicted.^6^, ^7^ We also excluded participants that have no arterial blood gases with a pH = 7.35–7.45 to avoid including those with acute respiratory acidosis.

Age, sex, race, and obstructive sleep apnea (OSA) (using ICD‐CM codes) were identified at the first COPD‐related hospitalization. Using chart review, we recorded the most recent pre‐bronchodilator FEV_1_%predicted and the most recent partial pressure of carbon dioxide (PaCO_2_) in an arterial blood gas with a pH = 7.35–7.45 between their first COPD‐related hospitalization and September 30, 2017. We recorded the most recent outpatient PaCO_2._ If outpatient PaCO_2_ was not available, we recorded the most recent inpatient PaCO_2_. We defined compensated hypercapnic respiratory failure (CompHRF) as PaCO_2_ > 45 mmHg in the most recent arterial blood gas.^8^ We used the term CompHRF as we cannot ensure that on the time of arterial blood gas measurement the patients were on chronic stable condition. CompHRF was used as a surrogate of CHRF. We also recorded the body mass index at the time of PaCO_2_. We reviewed discharge summaries to identify whether patients received mechanical ventilator support during COPD‐related hospitalizations and clinical notes to identify home NIV initiation using the following terms: AVAPS (average volume‐assured pressure support), bilevel, BIPAP, BPAP, NIV, pressure support, PS, and trilogy. We categorized patients by CompHRF and OSA and we compared their characteristics using chi‐squared of Fisher's exact for categorical variables and *t*‐test or Wilcoxon rank‐sum test for continuous variables.

## 3 RESULTS

Of 217 patients with at least one COPD‐related hospitalizations to the Iowa City VA Medical Center, we excluded 20 patients with no available spirometry or obstructive ventilatory defect and 11 patients that had no arterial blood gases with a pH = 7.35–7.45. Table 1 shows the characteristics of the remaining 186 individuals. The overall prevalence of CompHRF was 52.7%, while the overall prevalence of home NIV was 4.3% (*n* = 8). Of 51 patients that received mechanical ventilator support during hospital stay, seven (13.8%) received home NIV, while only one (0.7%) received home NIV among 135 patients that did not require inpatient mechanical ventilator support.

**TABLE 1:** Patients' characteristics stratified by number of COPD‐related hospitalizations.

Hospitalizations	All participants	1	2	3	≥4
Number	186	110	44	14	18
Age, years	67.0 [63.0, 73.8]	67.5 [63.0, 74.0]	67.5 [63.5, 73.3]	66.0 [61.8, 74.3]	66.5 [62.0, 70.0]
Male	177 (95.2%)	105 (95.5%)	40 (90.9%)	14 (100.0%)	18 (100.0%)
White	177 (95.2%)	105 (95.5%)	43 (97.7%)	13 (92.9%)	16 (88.9%)
Body mass index	28.9 [24.5, 34.4]	28.9 [24.3, 32.8]	28.8 [24.4, 40.1]	27.5 [23.4, 30.3]	30.6 [27.4, 34.5]
Pre‐FEV_1_%	45.0 [33.0, 56.0]	47.5 [34.3, 60.0]	48.0 [36.0, 56.0]	40.2 [28.3, 48.8]	33.0 [24.0, 39.0]
Pre‐FEV_1_ < 50%	115 (62.2%)	62 (56.4%)	26 (60.5%)	11 (78.6%)	16 (88.9%)
Outpatient ABG	119 (64.0%)	68 (61.8%)	28 (63.6%)	10 (71.4%)	13 (72.2%)
PaCO_2_, mmHg	44.9 [40.1, 53.9]	43.1 [39.5, 51.7]	47.1 [42.3, 54.9]	48.5 [44.9, 56.3]	51.1 [44.6, 56.2]
PaCO_2_ > 45 mmHg	98 (52.7%)	48 (43.6%)	25 (56.8%)	11 (78.6%)	14 (77.8%)
PaCO_2_ > 52 mmHg	53 (28.5%)	27 (24.5%)	14 (31.8%)	4 (28.6%)	8 (44.4%)
Inpatient mechanical ventilation support	51 (27.4%)	22 (20.0%)	12 (25.0%)	8 (57.1%)	9 (50%)
Home NIV	8 (4.3%)	2 (1.8%)	3 (6.8%)	1 (7.1%)	2 (11.1%)
Obstructive sleep apnea	65 (34.9%)	33 (30.0%)	18 (40.9%)	4 (28.6%)	10 (55.6%)
Death	68 (38.4%)	41 (39.4%)	13 (31.7%)	8 (57.1%)	6 (33.3%)

*Note*: Continuous variables are presented in median (IQI = interquartile interval).

Abbreviations: NIV, noninvasive ventilation; PaCO_2_, partial pressure of carbon dioxide in arterial blood; Pre‐FEV_1_%, pre‐bronchodilator FEV_1_% predicted.

The prevalence of CompHRF was 43.6%, and home NIV was 1.8% in those with one COPD‐related hospitalization. Among those with ≥4 COPD‐related hospitalizations, the prevalence of CompHRF was 77.8% (14 of 18), and home NIV was 11.1% (2 of 18).

Table 2 shows the characteristics in patient with and without CompHRF. The prevalence of NIV was 8.2% ( of 98) in patients with CompHRF while was 0 in those with no CompHRF. The median hospitalizations in patients with CompHRF was 2 (interquartile range [IQI]: 1–2.8), while the median hospitalizations in those with no CompHRF was 1 (IQI: 1–2; *P* value = 0.001).

**TABLE 2:** Patients' characteristics stratified by compensated hypercapnic respiratory failure.

Hospitalizations	No hypercapnia	Compensated hypercapnic respiratory failure	*P* value^a^
Number	88	98	
Age, years	68.0 [62.8, 74.0]	67.0 [63.0, 72.0]	0.53
Male	82 (93.2%)	95 (96.9%)	0.40
White	86 (97.7%)	91 (92.9%)	0.23
Body mass index	28.7 [24.9, 33.7]	29.0 [24.4, 34.5]	0.61
Pre‐FEV_1_%	53.5 [44.0, 64.3]	36.0 [28.0, 47.0]	<0.001
Pre‐FEV_1_ < 50%	38 (43.2%)	77 (79.4%)	<0.001
Outpatient ABG	57 (64.8%)	62 (63.3%)	0.88
PaCO_2_, mmHg	40.1 [37.3, 42.9]	52.6 [47.3, 62.0]	<0.001
Home NIV	0 (0.0%)	8 (8.2%)	0.017
Obstructive sleep apnea	25 (28.4%)	40 (40.8%)	0.106
Hospitalizations	1.0 [1.0, 2.0]	2.00 [1.0, 2.8]	0.001
Death	26 (31.0%)	42 (45.2%)	0.074

*Note*: Continuous variables are presented in median (IQI = interquartile interval).

Abbreviations: NIV, noninvasive ventilation; PaCO_2_, partial pressure of carbon dioxide in arterial blood; Pre‐FEV_1_%, pre‐bronchodilator FEV_1_% predicted.

^a^ We compared the characteristics between patients with and without compensated chronic hypercapnic respiratory failure using chi‐squared of Fisher's exact for categorical variables and t‐test or Wilcoxon rank‐sum test for continuous variables.

Table 3 shows the characteristics in patients with and without OSA. Patients with OSA had higher body mass index and PaCO_2_ levels relative to those without OSA.

**TABLE 3:** Patients' characteristics stratified by obstructive sleep apnea.

Hospitalizations	No obstructive sleep apnea	Obstructive sleep apnea	*P* value^a^
Number	88	98	
Age, years	68.0 [64.0, 74.0]	66.0 [61.0, 73.0]	0.051
Male	115 (95.0%)	62 (95.4%)	1
White	117 (96.7%)	60 (92.3%)	0.33
Body mass index	26.97 [23.4, 30.8]	34.41 [28.7, 41.1]	<0.001
Pre‐FEV_1_%	47.0 [33.0, 56.0]	42.2 [32.8, 54.3]	0.65
Pre‐FEV_1_ < 50%	74 (61.2%)	41 (64.1%)	0.82
Outpatient ABG	82 (67.8%)	37 (56.9%)	0.15
PaCO_2_, mmHg	43.1 [39.2, 53.1]	46.5 [43.3, 55.8]	0.014
Home NIV	3 (2.5%)	5 (7.7%)	0.20
Hospitalizations	1.0 [1.0, 2.0]	1.0 [1.0, 2.0]	0.067
Death	42 (37.2%)	26 (40.6%)	0.77

*Note*: Continuous variables are presented in median (IQI = interquartile interval).

Abbreviations: NIV, noninvasive ventilation; PaCO_2_, partial pressure of carbon dioxide in arterial blood; Pre‐FEV_1_%, pre‐bronchodilator FEV_1_% predicted.

^a^ We compared the characteristics between patients with and without obstructive sleep apnea using chi‐squared of Fisher's exact for categorical variables and t‐test or Wilcoxon rank‐sum test for continuous variables.

## 4 DISCUSSION

The prevalence of CHRF in the entire COPD population is unknown. In the general population, a recent study showed that CHRF due to any causes, but not necessarily COPD‐related CHRF, ranged from 0.08% in 45‐ to 54‐year‐old individuals to 1.7% in those aged over 85 years.^9^ A German study showed 9% of patients with severe lung function impairment have PaCO_2_ > 50 mmHg.^10^ This pilot study in a single VA hospital showed that approximately half of patients with COPD‐related hospitalizations have CompHRF. CompHRF prevalence increases with increasing number of COPD‐related hospitalizations. A small prospective study in two medical centers in China showed a 43% prevalence of CHRF among those hospitalized.^11^

Home NIV use in patients with CHRF due COPD is associated with decrease in mortality and hospitalizations.^1^, ^2^, ^3^ Despite the significant benefit of home NIV for CHRF, our findings showed that home NIV use is only 8.2% among patient with CompHRF and 4.3%(8 of 186) among all individuals with COPD‐related hospitalizations, which is almost double the prevalence of home NIV use in a prior study.^4^ This is likely due to fact that our study was more recent.

The underutilization of home NIV in COPD has been shown in other reports.^4^, ^12^ A Canadian study using administrative data of 13 000 000 people found that 4670 used home NIV for any indication (e.g., neuromuscular disease). Of those patients with home NIV, only 18.8% had COPD despite being the most common cause of CHRF.^12^

Our study has several limitations including that we do not have information regarding the condition of the participants when the PaCO_2_ was obtained. We excluded patients with no available spirometry data to ensure that we only captured patients with CHRF due to COPD. Spirometry data were not recorded necessary at the same time as PaCO_2_. Our sample size was small, and we conducted the study in a single VA hospital that included predominantly males. We do not have data regarding smoking exposure, post‐bronchodilator spirometry, and oxygen use. Participants in our cohort may have CHRF due to obesity hypoventilation syndrome as 34.9% had concomitant OSA.

In conclusion, approximately half of individuals with at least one COPD‐related hospitalization have CompHRF, but only 8.2% of those use home NIV. Future studies should estimate CHRF rates and the degree of underutilization of home NIV in larger multicenter samples and identify barriers and facilitators for home NIV use in patients with COPD‐related hospitalizations who are at high‐risk for CHRF.

## AUTHOR CONTRIBUTIONS

Spyridon Fortis is responsible for the concept and the design of the study and had full access to all data in the study and take responsibility for the integrity of the data and the accuracy of the data analysis. All authors contributed to data collection, analysis, drafting, and revising and gave the approval of the final version to be published.

## CONFLICT OF INTEREST STATEMENT

Dr. Fortis has received grants from American Thoracic Society and Fisher & Paykel and has consulted Society of Hospital Medicine.

## DISCLAIMER

The views expressed in this article are those of the authors and do not necessarily reflect the position or policy of the Department of Veterans Affairs or the US Government and of the National Institutes of Health's National Center for Advancing Translational Sciences.

## ETHICS STATEMENT

The Institutional Review Board and Research and Development Committee at the Iowa City VA [IRB 201712713] approved this study.

## Data Availability

The data that support the findings of this study are available from the US Department of Veterans Affairs (VA) but restrictions apply to the availability of these data, which were used under license for the current study, and so are not publicly available. Data are however available from the authors upon reasonable request and with permission of VA. Original VA‐funded datasets will be retained on VA servers behind VA firewalls. These data will be provided to interested parties following proper filing and verification of a Freedom of Information Act (FOIA) request and approval by the Privacy Officer. These data will be maintained as required by VA data retention policies. Maintenance of original datasets and/or programming code to create analytical datasets from large, centralized VA data sources will permit validation of results.
